# *Mycobacteriumtuberculosis* antigens repress Th1 immune response suppression and promotes lung cancer metastasis through PD-1/PDl-1 signaling pathway

**DOI:** 10.1038/s41419-018-1237-y

**Published:** 2019-01-18

**Authors:** Shuhui Cao, Jingwen Li, Jun Lu, Runbo Zhong, Hua Zhong

**Affiliations:** Department of Pulmonary, Shanghai Chest Hospital, Shanghai Jiaotong University, Shanghai, 200030 China

## Abstract

Given one-third of the world’s population is infected with *Mycobacterium* *tuberculosis* (MTB), it is important to identify the underling molecular mechanism between development of TB and lung cancer. This study investigated the immune response to MTB infection on lung metastasis in lung cancer cells via T cell-mediated immune response. To clarify this problem, we analyzed the expression levels of PD-1, PD-L1, and PD-L2 and immune function in antigen-specific T cell as derived from MTB patients or spleen lymphocytes derived from wild-type and PD-1 knockout mice with MTB antigen stimulation and Lewis lung cancer cells injection. Our data indicate that the expression levels of PD-1, PD-L1, and PD-L2 were elevated in active pulmonary TB patients, as well as in mice received MTB and lung cancer cells treatment. We also observed the T cell-mediated cellular immune response were inhibited by MTB while MTB significantly promote tumor metastasis in lung. In conclusion, the PD-1/PD-L pathway is required MTB repressed T-cell immune response and promotes tumor metastasis. This study provides evidence that blockade of PD-1/PD-L1 signaling pathway may benefit patients with MTB or other chronic infection and even prevent them from development of cancer.

## Introduction

Lung cancer is by far the leading cause of death from cancer, with an estimated 1.59 million people dying from lung cancer in 2016, accounting for approximately 20% of all cancer deaths worldwide^1^. The incidence and mortality of lung cancer have been increasing rapidly in China, making lung cancer the first leading cause of cancer death since 2010 and an emerging health issue in the country^2^. Therefore, an up-to-date study of epidemiology of lung cancer in China, including smoking, air pollution, occupational risk factors, would provide the evidence base for future interventions to improve this health issue in China^3^.

Mycobacterium tuberculosis (MTB) is the pathogen that causes tuberculosis (TB), which is now the world’s deadliest infectious diseases^4^. One-third of the world’s population is infected by MTB, while 5–10% of infected people will develop TB if the treatment is inadequate, or if host defenses are impaired. Most MTB infections do not have any symptoms and TB-induced inflammation often eventually lead to genetic change and even lung cancer. On the other hand, increased lung cancer incidence is related to immunosuppression status resulted from MTB infection^5,6^. Concurrent TB and lung cancer were reported in a large number of cases and case–control studies^7–9^.

In the early stage of MTB infection, activation of immune response with type 1T helper cells (Th1) and production of IFN-γ and TNF-α are most prominent protective mechanism for intracellular mycobacterial killing. An important process in T cell-mediated immune response is the interaction between co-stimulatory and co-inhibitory receptors on T-cell surface (e.g., CD28 and CTLA-4) and CD80 (B7-1) and CD86 (B7-2) presented on antigen presenting cells (APCs). It is also believed inhibitory mechanisms such as immune evasion and immune checkpoint inhibition are involved to allow MTB to establish latent infections^10^.

Lately a few studies have shown that PD-1-PD-L1 pathway impairs Th1 immune response in late stage of infection, which implicates the inhibitory PD-1/PD-L1 pathway with the functional impairment of T cells^11,12^. Blocking PD-1-PD-L1 signaling pathway is reported to successfully restore T-cell function in lymphoma, showing the effectiveness of PD-1/PD-L1 blockade therapy for various malignancies, including lung cancer^13,14^. The goal of this study is to improve understanding of the immune regulatory mechanism in MTB infection, as well as enhance the development of potential PD-1/PD-L1 blockade to overcome the resistance mechanisms in TB disease, and to combat lung cancer.

## Material and method

### Study subjects

Five patients with pulmonary TB were enrolled from the Thoracic hospital affiliated to Shanghai Jiaotong University, which was approved by the Institutional Review Board of the hospital. Informed consent was signed and provided. TB infection was diagnosed on the basis of clinical findings and supporting evidence from ancillary tests such as lung imaging and sputum Gram’s staining. Five healthy individuals vaccinated with BCG vaccine for tuberculosis were included as control. Peripheral blood was collected from all subjects to isolate peripheral blood mononuclear cells. All patients received anti-TB treatment after blood draw.

### Soluble antigen stimulation

Circulating human peripheral blood mononuclear cells (PBMCs) as well as mice spleen lymphocytes were isolated by Ficoll-paque (Amersham biosciences) density gradient centrifugation. A total of 1 × 10^6^ cells were cultured in 24-well plate (Cellstar, Greiner Bio-one) with RPMI1640 medium supplemented with 10% human serum, 2mM l-glutamine (Sigma-Aldrich), 100 µ/ml penicillin, and 100 μg/ml streptomycin. The human PBMCs were stimulated by MTB antigen (10 mg/ml) for 5 days while the mic spleen lymphocytes were stimulated by Lewis lung carcinoma cells (LLC) soluble antigen (10 mg/ml) for 5 days. After treatment, both cells were analyzed by with flow cytometry to evaluate the ratio of PD-1 and PD-L1 positive cells.

LLC were cultured in high glucose DMEM medium supplemented with 10% FBS, 2mM l-glutamine (Sigma-Aldrich), 100 µ/ml penicillin, and 100 μg/ml streptomycin. After ddH_2_O resuspension and four times of repeated freezing and thawing, the LLC cells were collected and stored at −80 °C.

### Bacteria

MTB strain BV173 were grown in Middlebrook 7H9 liquid medium with 0.2% glycerol and ADC (0.5% bovine serum albumin, 0.2% glucose, 3 μg/ml catalase). When in mid-log phase bacterial stocks were collected, separated, and frozen in 1 ml aliquots at –80 °C. A few days later, aliquots of MTB stocks were thawed and used to control for bacterial load. For this, serial dilutions of 6 frozen vials of each strain were plated in 7H11 agar plates and viable bacteria (colony forming units) counted after 3 weeks of incubation at 37 °C. The obtained value was used to calculate the concentration of each strain stock used.

### Co-stimulation mice model

C57BL/6 control mice and PD-1 knockout (PD-1 KO or PD-1^−/−^) C57BL/6 mice were obtained from Jackson laboratory. For all experiments 3-week-old male mice were used as approved by the Animal Ethics Committee of the Thoracic hospital affiliated to Shanghai Jiaotong University. To develop lung cancer and MTB co-stimulation mice model, the mice were received two stimulations step by step. At first, one group mice were injected with 1 × 10^6^ CFU BV173 bacteria, and the other group of mice were only administrated with PBS as control. Next, both two groups of mice were injected with 2 × 10^6^ LLC cells through tail vein, and executed in 7, 14, 21, and 30 days after LLC cells injection. The mice spleen lymphocytes were isolated to measure the expression level of PD-1 and PD-L1 on the surface of tumor antigen-specific T cells and the response of tumor-specific Th1 cells. Spleen lymphocytes from 21 days after LLC cell injection were used in cell proliferation assay. In addition to that, the lung tissue were collected in 30 days after LLC cells injection, fixed by Bouin’s solution. The pulmonary metastasis nodule was examined and counted under the microscope.

### BMDC and lymphocytes co-culture cell model

The co-culture cell model includes lymphocytes from either C57BL/6 control mice or PD-1 KO mice and bone marrow derived cells (BMDCs). BMDCs were cultured in RPMI1640 medium supplemented with 10% FBS, 2 mM glutamine, 100 µ/ml penicillin, and 100 μg/ml streptomycin while the medium was replaced every 3 days. After 8-day culture, the LLC cell lysis (10 mg/ml) was added in the medium for 16 h, followed with 50 μg/ml mitomycin C treatment for 30 min. Next, the 5 × 10^5^/ml BMDCs were co-cultured with the spleen lymphocytes (1 × 10^6^/ml, from mice treated with LLC for 21 days) for 60 h. Cell numbers were counted to generate cell growth curve via cell counting kit (Sigma-Aldrich). The CBA Mouse Inflammation kit (BD Biosciences) was used to measure IFN-γ and TNF-α levels in the supernatant while flow cytometry was used to measure IFN-γ and TNF-α level expressed on CD4+ T cells.

LDH cytotoxicity Assay Kit (Pierce Biotechnology) was used to assay for cytotoxicity in co-cultured spleen lymphocytes following the manufacturer instructions. Briefly, the spleen lymphocytes and LLC were seeded in a ratio of 50:1 in 96-well plate after co-cultured with BMDCs, and cultured for another 24 h. The percentage of cell viability was calculated as follows: the % cell cytotoxicity = [(experimental value − effector cells spontaneous control − target cells spontaneous control)/target cell maximum control − target cells spontaneous control]×100%. All calculations were performed after background absorbance correction and blank absorbance subtraction.

### Flow cytometry

Cell flow cytometry analysis was conducted to measure CD3, CD4, CD8, and PD-1 expression levels in human PBMCs as stimulated by MTB antigen. The mouse spleen lymphocytes were analyzed after stained by fluorescein isothiocyanate (FITC) labeled 1-A^b^ antbody (or CD44), phycoerythrin (PE)—Cy5 labeled CD4 antobody, as well as PE labeled B7-1, B7-2, HVEM, ICOS-L, PD-1, PD-L1, CD62L, and control antibodies. The cells were subsequently acquired with FACSCalibur and analyzed with CellQuest (BD Biosciences) or Flowjo (Tree Star) software. Apoptosis of CD4+ T cells was determined by flow cytometry using Annexin V-PE dual staining (Calbiochem). FOXP3 staining in CD4+ T cells was performed by FITC anti-FOXP3 staining kit (eBioscience).

### Statistical analysis

The significance of the results was determined using Student’s *t* test. *P* values of <0.05 were considered significant. Results are presented graphically by column diagrams. All data are provided as mean ± SD.

## Result

### Expression of PD-1, PD-L1, and PDL-2 is increased on human T cells after stimulated by MTB antigen

**Fig. 1:**
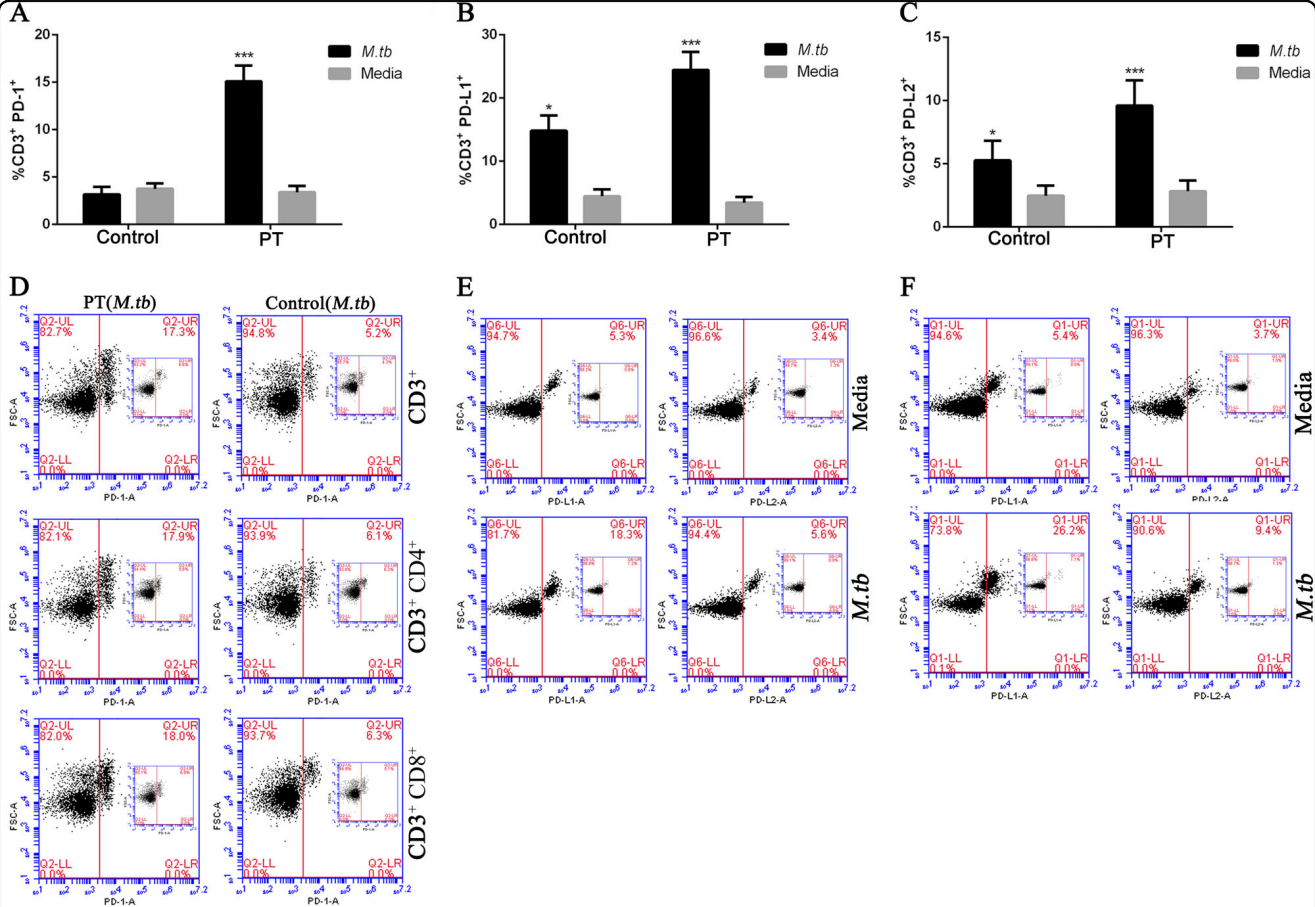
Enhanced expression of PD-1 and its ligands on T cells from pulmonary tuberculosis patients. **a**–**c** Representative bar graphs showing the expression levels of PD-1 (**a**), PD-L1 (**b**) and PD-L2 (**c**) in CD3+ cells from control and pulmonary tuberculosis (PT) patients, with or without MTB antigen (M. tb) stimulation (^*^p < 0.05, ^***^p < 0.001, M. tb vs. media); **d** representative flow cytometry data showing expression levels of PD-1 in total CD3+ cells, CD4+/CD3+, and CD8+/CD3 + cells with MTB antigen stimulation; **e** the expression levels of PD-L1 and PD-L2 in CD3+ cells from control subjects; and **f** the expression level of PD-L1 and PD-L2 in CD3+ cells from pulmonary tuberculosis patients

To investigate if MTB antigen stimulation could activate PD-1 signaling pathway in human immune cells from pulmonary TB patients, flow cytometry was performed to detect PD-1 and its two ligands (PD-L1 and PDL-2) expression on CD3+ T cells collected from the subjects. We observed significantly increased expression levels of PD-1 (Fig. 1a, d), as well as its two ligands PD-L1 and PDL-2 (Fig. 1b, c, e, f) on CD3+ T cells from of pulmonary TB patients but not in control subjects. Moreover, the expression level of PD-1 in both CD4+/CD3+ and CD8+/CD3+ T cells were constantly increased as it was in total CD3+ T cells. Together, this result indicate MTB antigen significantly activate PD-1 signaling pathways to drive both CD4 and CD8 T-cell immune response.

### Expression of PD-1 and PDL-1 is increased on mice T cells after co-stimulated by tumor antigen and MTB antigen

**Fig. 2:**
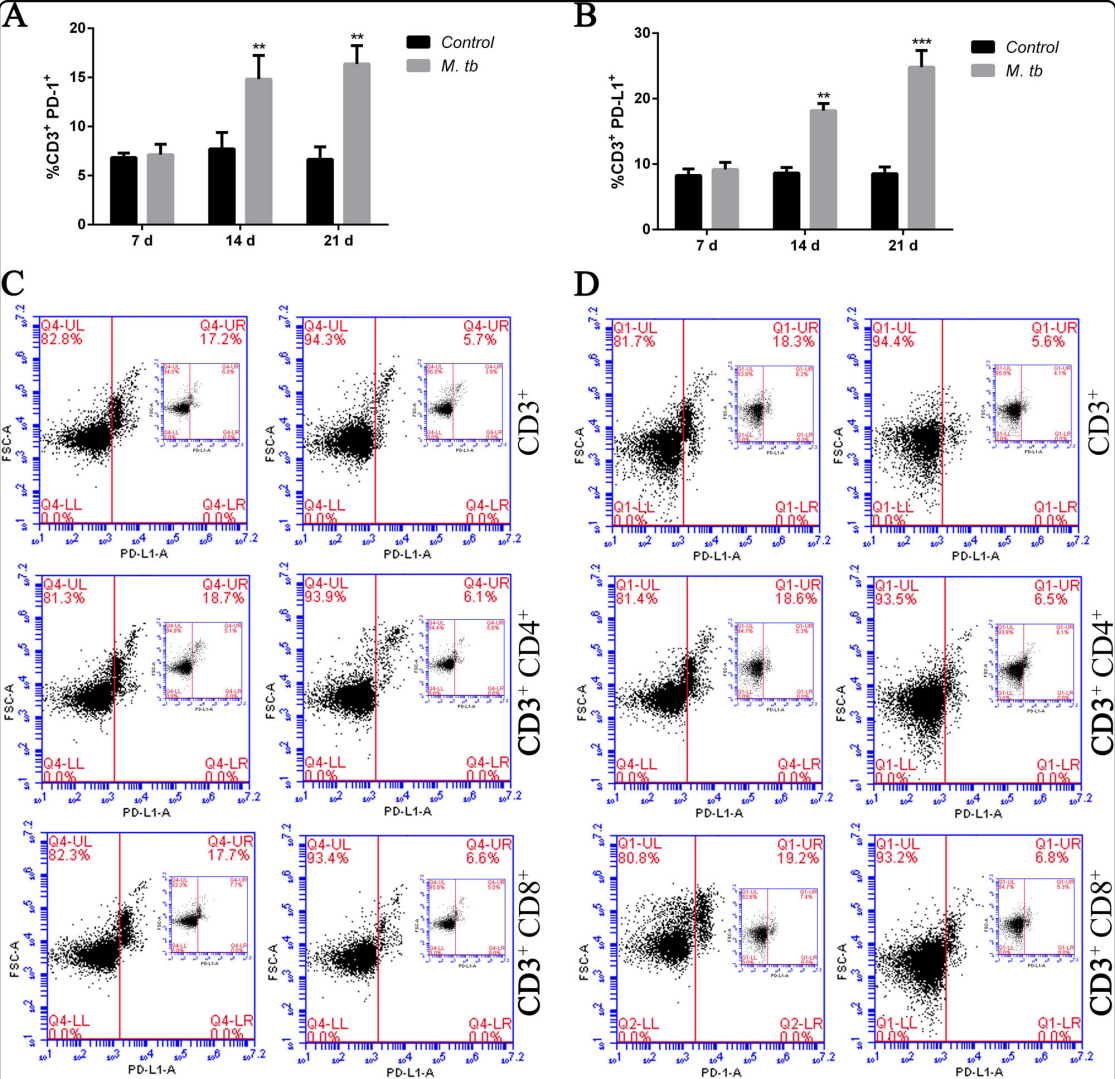
Enhanced expression of PD-1 and its ligands on T cells derived from co-stimulated mice. **a**–**b** Representative bar graphs showing the expression level of PD-1 (**a**) and PD-L1 (**b**) on CD3+ T cells at different time points (7, 14, and 21 days) after LLC cell injection, with or without MTB antigen (M. tb) stimulation (^**^p < 0.01, ^***^p < 0.001, M. tb vs. control); **c**–**d** twenty-one days after LLC injection, representative flow cytometry data showing the expression levels of PD-1 (**c**) and PD-L1 (**d**) in total CD3+ cells, CD4+/CD3+, and CD8+/CD3+ cells with or without MTB antigen stimulation

To further investigate if the PD-1 signaling could be activated by combination of MTB antigen as well as tumor antigen from lung cancer, we generated a co-stimulation mice model and analyzed the lymphocytes. The LLC cell line from a Lewis lung tumor originated obtained from the lung of a C57BL mouse was used as a source of lung tumor antigen. As showed in Fig. 2a, c, PD-1 expression on the CD3+ T cell from MTB infected mice was time-dependent increased. MTB antigen significantly induced PD-1 expression after 14 and 21 days LLC injection but not in earlier time point (7 days). PD-L1 expression showed similar increase in a time-dependent manner (Fig. 2b, d). These data confirmed lung tumor antigen stimulation is required for MTB induced PD-1 signaling activation in T-cell immune response.

### PD-1 is required for MTB antigen mediated cellular immune response

**Fig. 3:**
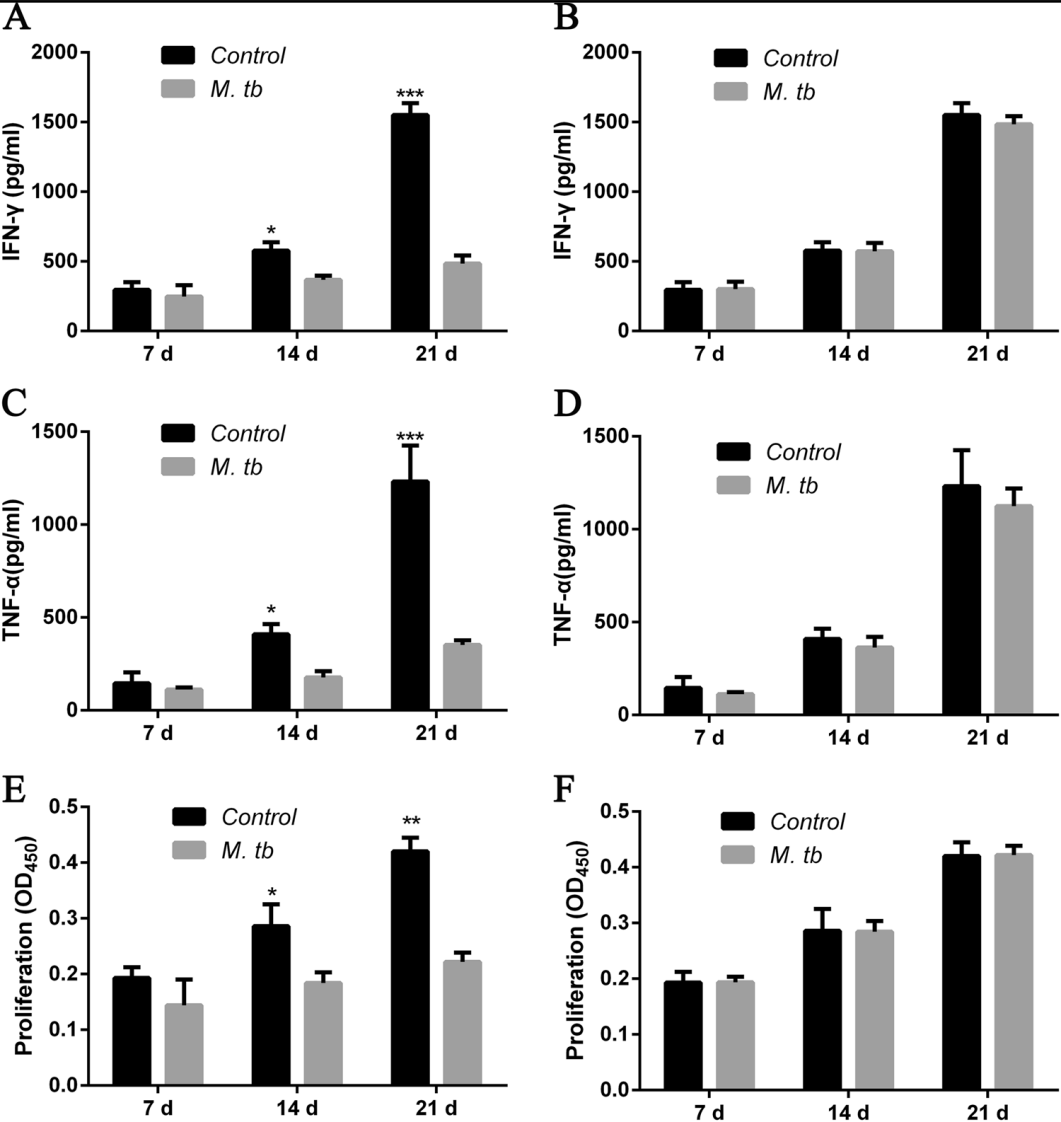
PD-1 is required for MTB mediated cellular immune cell response. **a**–**b** Representative flow cytometry data showing IFN-γ levels in wild-type (**a**) and PD-1 KO (**b**) T cells at different time points (7, 14, and 21 days) after LLC cell injection, with or without MTB antigen (M. tb) stimulation (^**^p < 0.01, ^***^p < 0.001, M. tb vs. control); **c**–**d** TNF-α levels in wild-type (**c**) and PD-1 KO (**d**) T cells at different conditions as indicated (^**^p < 0.01, ^***^p < 0.001, M. tb vs. control); and **e**–**f** cell proliferation of T cells in various condition (as indicated) measured by CCK-8 kit stimulation (^*^p < 0.05, ^**^p < 0.01, M. tb vs. control)

**Fig. 4:**
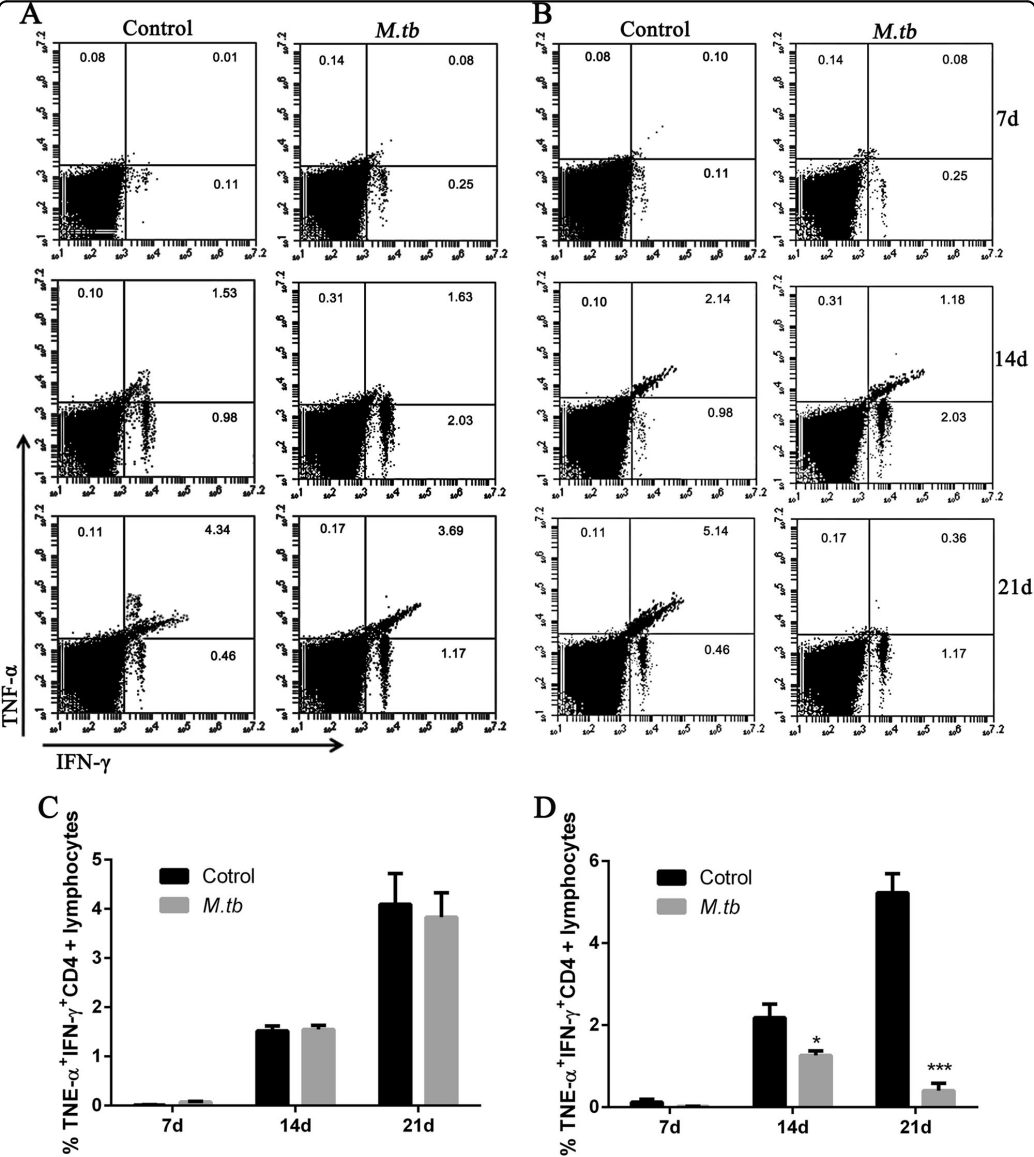
MTB antigen mediated inhibition of IFN-γ and TNF-α is dependent on PD-1. **a** Representative flow cytometry data showing the percentage of IFN-γ and TNF-α stained PD-1 KO T cells at different time points (7, 14, and 21 days) after LLC cell injection, with or without MTB antigen (M. tb) stimulation and **b** the percentage of IFN-γ and TNF-α stained wild-type T cells at different conditions as indicated; **c**-**d**: populations of TNF-α+IFN-γ+CD4+lymphocytes in PD-1 KO mice and wild-typemice (as indicated)

To explore weather MTB induced immune response requires PD-1, mice BMDCs were isolated from LLC injected mice and co-cultured with lymphocytes from either C57BL/6 control mice (wide-type) or PD-1 KO mice. The lymphocytes were analyzed directly or sorted out to evaluate the cellular immune response. We firstly detected IFN-γ and TNF-α levels in CD4+ T cells as isolated from the co-cultured lymphocytes. The results showed that the IFN-γ and TNF-α levels were significantly reduced in MTB stimulated wild-type CD4+ T cells (Fig. 3a, c) but not in PD-1 KO CD4+ T cells (Fig. 3b, d). Comparing ratio of IFN-γ and TNF-α levels showed very clearly that less and less IFN-γ and TNF-α protein were detected in MTB antigen treated wild-type T cells (Fig. 4b right), compared to PD-1 KO T cells (Fig. 4a right) or T cells without MTB antigen treatment (Fig. 4a left, 4B left). Cell proliferation assay also showed MTB antigen only inhibited cell growth in wild-type CD4+ T cells (Fig. 3e) but not in PD-1 KO CD4+ T cells (Fig. 3f). Interestingly, both cytokine releasing and cell proliferation were induced following LLC injection in time-dependent manner.

**Fig. 5:**
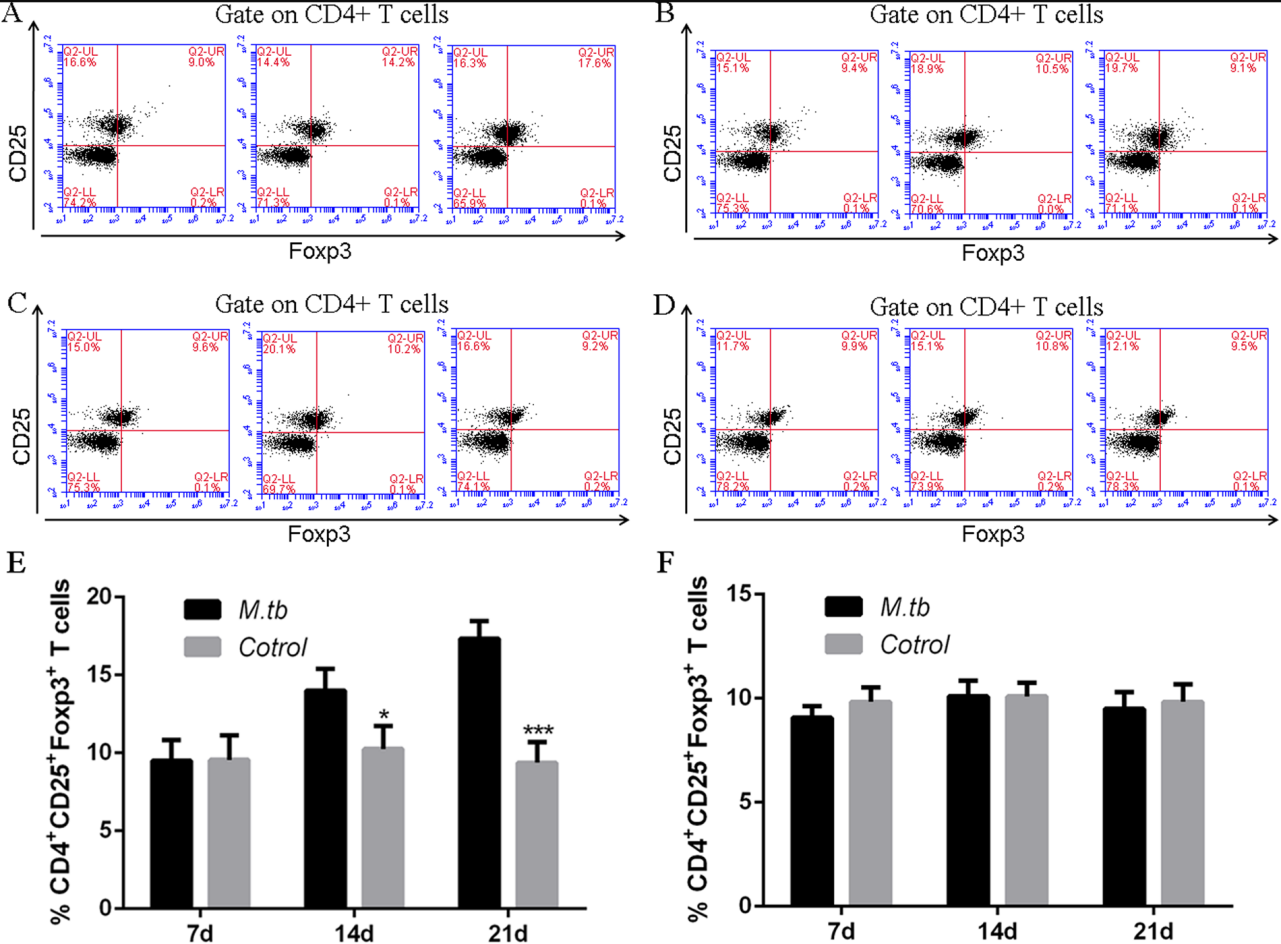
CD4+ CD25+ Foxp3+ T cells (Tregs) with MTB antigen stimulation responds to LLC cell injection. **a**–**b** Representative flow cytometry data for the percentage of wild-type Tregs cells at different time points (7, 14, and 21 days) after LLC cell injection, with (**a**) or without MTB antigen (M. tb) (**b**) stimulation and **b** the percentage of PD-1 KO Tregs cells at different time points as indicated, with (**c**) or without MTB antigen (M. tb) (**d**) stimulation. **e**–**f**: populations of CD4+CD25+Foxp3+T cells in wild-type mice and PD-1 KO mice (as indicated)

**Fig. 6:**
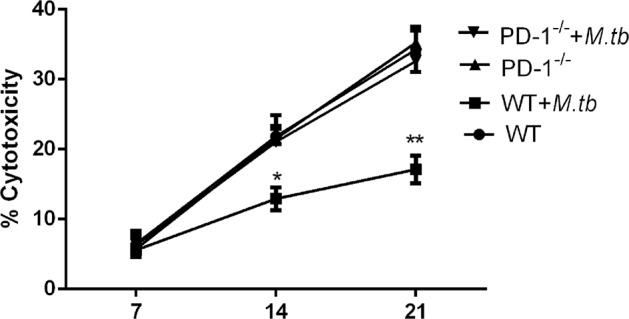
LDH-releasing assay detected reduced cytotoxicity of CD8+ T cells. Reduced cytotoxicity of effector CD8+ T cells was detected in MTB antigen stimulated wild-type T cells (^*^p < 0.05; ^**^p < 0.01)

To further evaluate the Tregs cell activation during MTB antigen mediated immune response, CD4+ CD25+ Foxp3+ T cells (Treg cell) were counted by flow cytometry. Similar to previous finding, only wild-type Treg cell with MTB antigen stimulation was increased during LLC cell injection (Fig. 5a). Wild-type Treg cell with no MTB antigen stimulation (Fig. 5b) or PD-1 KO Tregs (Fig. 5c, d) showed no difference during LLC cell injection. Moreover, the LDH levels in the supernatants from those cells were measured to evaluate the cytotoxicity of CD8+ T cells. LDH cytotoxicity assay suggested cytotoxicity of CD8+ T cells is weakened by MTB antigen only in wild-type T cells but not in PD-1 KO T cells (Fig. 6). Together, we concluded PD-1 is required for MTB antigen repressed T-cell immune response.

### APCs and CD4+ memory T cells from mice stimulated with MTB antigen and LLC cells are dysfunctional

Activation of a T cell by antigen requires presentation of antigen by MHC on APCs and the interaction between B7 on the APCs and CD28/CTLA-4 on the membrane of the T cell. The repressed T-cell immune response observed above may be associated with the expression of ligand of the CD28/CTLA-4 receptor family on APC. To test this hypothesis, we examined the expression levels of multiple ligands on MHC class II^high^ (I-A^high^) APCs (including DC and macrophages).

**Fig. 7:**
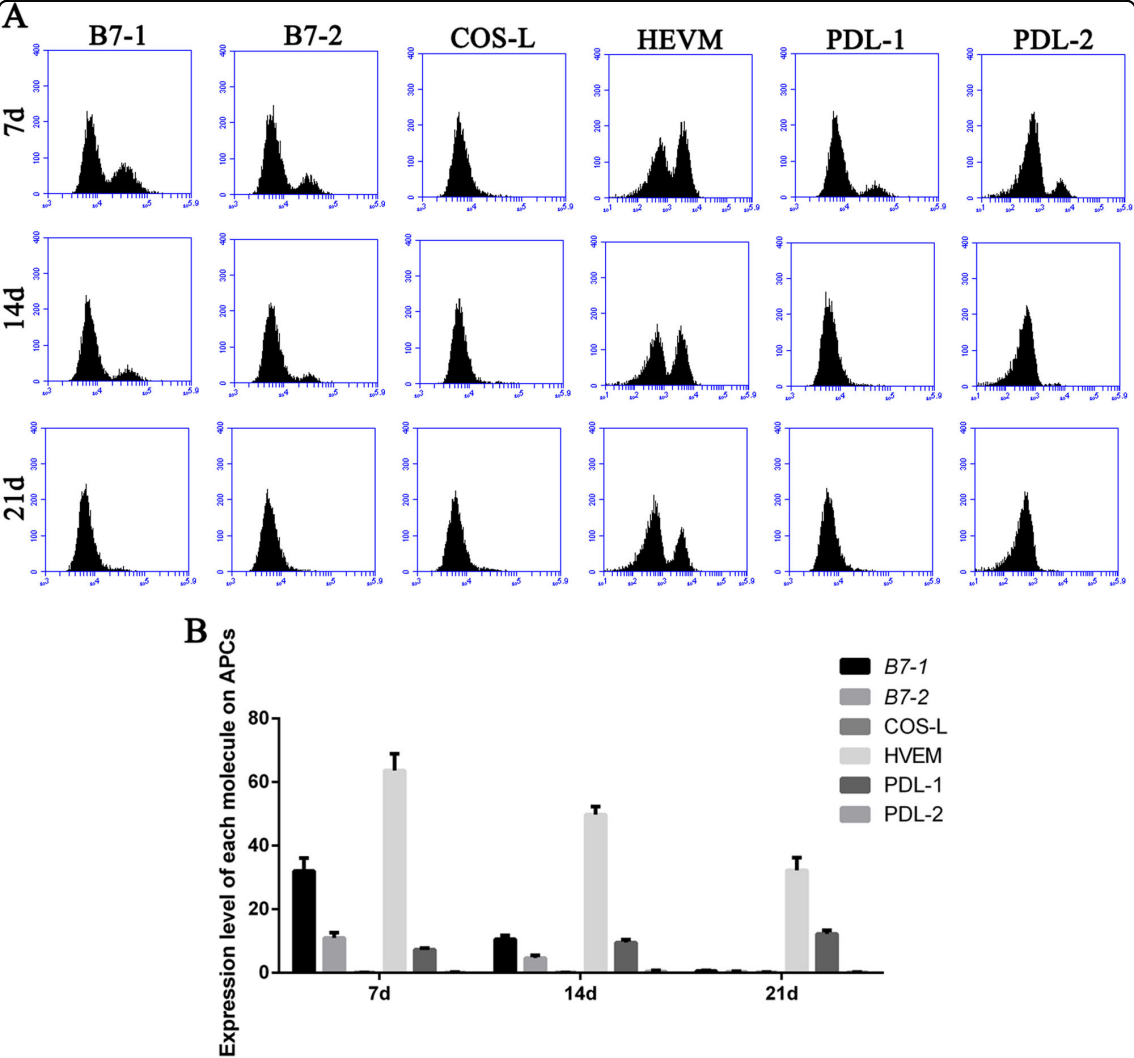
Expression of ligand of CD28/CTLA-4 receptor family was changed in APCs. **a**-**b**: Representative flow cytometry data for the expression levels of B7-1 (CD80), B7-2 (CD86), HVEM, ICOS-l, PD-L1, and PD-L2 in APCs from MTB antigen stimulated wild-type mice with LLC cell injection at different time points

The expression levels of B7-1 (CD80), B7-2 (CD86), and HVEM in APCs derived from MTB infected wild-type mice were decreased during LLC cell injection, the expression levels of ICOS-l and PD-L2 remained the same while PD-L1 expression was increased in response to LLC cell injection (Fig. 7). Considering previous data indicated that MTB antigen and LLC cells co-stimulation induced PD-1 expression in T cells, our data is in agree with the hypothesis that PD-1/PDL-1 signaling pathway play an important role in the T-cell immune response.

**Fig. 8:**
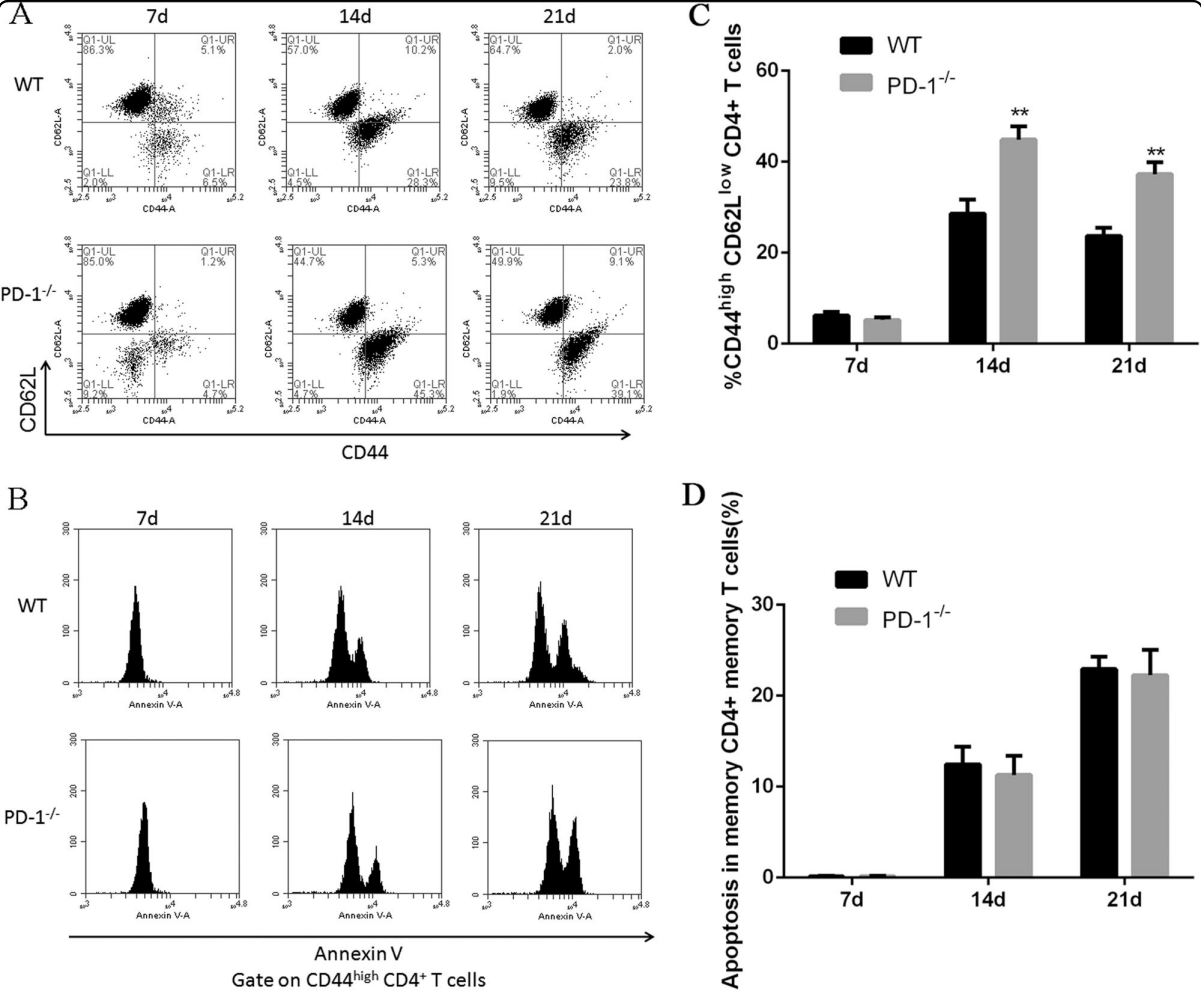
MTB antigen stimulated CD4+ memory T cells activation was inhibited in response to LLC cell injection. **a and c** Representative flow cytometry analysis of the percentage of CD4+ memory T cells (CD44^high^ CD62L^low^ CD4+) from MTB antigen stimulated wild-type mice and PD-1 KO mice and **b and d** analysis of cell apoptosis in memory CD4+ memory T cells as indicated

In addition to that, we found that the percentage of CD4+ memory T cells (CD44^high^ CD62L^low^ CD4+) in stimulated wild-type mice was significantly lower than PD-1 KO mice. The apoptosis of CD4+ memory T cells was analyzed by Annexin V staining in flow cytometry. Notably, no significant different apoptosis was noticed between CD4+ memory T cells derived from wild-type mice versus PD-1 KO mice, indicating that the PD-1/PDL-1 signaling pathway may inhibit the production of memory T cells (Fig. 8).

### MTB infection promotes in vivo lung metastases of lung cancer cells

**Fig. 9:**
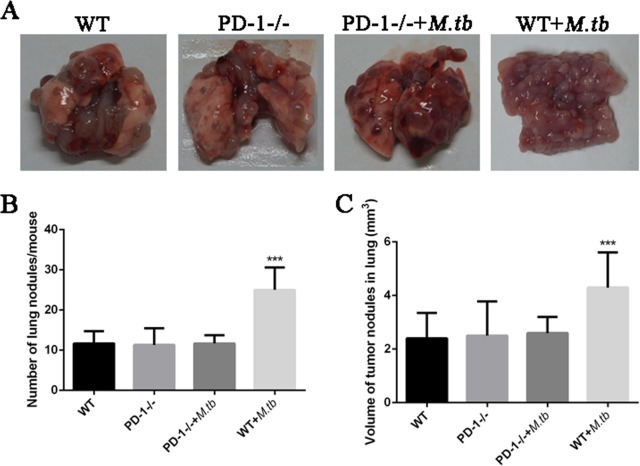
MTB antigen stimulation promotes macroscopically detected lung metastases. **a** Representative of lung tissue collected from wild-type and PD-1 KO mice with or without MTB antigen stimulation; **b**–**c** the average number and volume of lung metastatic nodules from wild-type and PD-1 KO mice with or without MTB antigen stimulation (^***^p < 0.001, M. tb vs. control)

To further confirm the effect of MTB infection on the metastatic potential of the lung cancer cells LLC in vivo, the MTB infection-mediated effect on lung metastases of LLC was assessed in the C57BL/6 mice. As shown in Fig. 9a, there was a significant difference of lung metastases between MTB treated and untreated lung from wild-type mice but no difference was observed in PD-1 KO mice. Similarly, the number (Fig. 9b) and volume (Fig. 9c) of lung metastatic nodules are significantly increased in MTB treated wild-type mice compared to untreated wild-type mice while this difference is diminished in PD-1 KO mice. This data suggested MTB infection enhances the tumor metastases in lung tissue, while PD-1 signaling is required in this regulation.

## Discussion

Our hypothesis was MTB infection-mediated immune response and its facilitated tumor metastases is associated with PD-1/PD-L1 signaling pathway. The present study has delineated clear differences of immune cells derived from PD-1 KO mice and wild-type mice. Indeed, we report for the first time the major reductions not only in total CD4 and CD8 T cell and it mediated cell immune response but also in Tregs and CD4 memory T cells subsets, in response to the co-stimulation from both MTB antigen and lung cancer antigen. We also identified PD-1 promotes lung metastasis in vivo. Thus this study identified previously unrecognized fact that regulation of PD-1 signaling pathway in TB patients may provide beneficial effect.

Many studies have shown that early pulmonary TB is an independent risk factor for the onset of lung cancer^15–18^. A cohort study showed that patients with pulmonary TB had a much higher incidence of lung cancer, with an adjusted HR of 3.32 during 7–9 years of follow-up, and a significant increase in mortality in patients with TB cancer^19^. Another study also showed that the incidence of lung cancer was higher in patients with new tuberculosis, and that the risk in seniors are increased while the incidence of males was higher than that of females^20^. The correlation between pulmonary tuberculosis and lung cancer was noticed in both men and women, and was independent with factors like smoking and indoor environmental conditions^17^. In agree with previous findings, our data provide insight that pulmonary TB antigen may collaborate with tumor antigen and inhibit immune responses.

The increased risk of lung cancer in TB patients is biologically plausible. Respiratory symptoms may have lasted for several months before TB was diagnosed, and TB treatment usually takes 6–9 months of combined drug therapy^9^. During this period, the infection causes severe lung inflammation, and the formation of tumor necrosis factor causes long-term persistent fever and weight loss^21^. Activated leukocyte participates in the inflammatory reaction of reactive oxygen and nitrogen oxides that can bind to DNA and lead to genomic mutation. More importantly, TB also causes extensive pulmonary fibrosis, which is associated with the production of TGF-Β, IL-4, and IL-13^22,23^. At present, the mechanism of increasing lung cancer risk of pulmonary TB is still unclear, and cellular immunization plays a decisive role in the development and progression of the tumor. Specific receptors on T lymphocytes recognize the antigens and release activation signals that induce the production of cytokines such IFN-γ. In consistent with that, our results showed cytokines IFN-γ and TNF-α levels were significantly increased during LLC cell injection and were significantly reduced after MTB stimulation. PD-1/PD-L1 acts important roles in this activation because this regulation was completely abolished in PD-1 KO mice.

The PD-1 axis is one of the key immunoregulatory pathways that mediate T-cell exhaustion in chronic infections. Activation of PD-1 and PD-L1 signaling pathways can lead to the formation of immunosuppressive tumor microenvironment, while blocking PD-1/PD-L1 signaling pathway may reverse tumor immune microenvironment and enhance endogenous anti-tumor immune effect^24,25^. The roles of PD-1 and PD-L1 signaling pathways in chronic infection such as TB infection is not clear. It was only recently researchers noticed a significant decline in PD-1, PD-L1 and PD-L2 gene expression in blood cells from TB patients while PD-1 protein expression CD8+ and CD4+ T cells was similar in patients with active TB disease compared to controls^26^. In comparison to that, our studies investigated MTB antigen infection could rapidly increase PD-1 and PD-L1 expression on the CD3+ T cells derived from human TB patients or LLC cell injected mice. In agree with our result, analysis on a Chinese cohort reported PD-1, PD-L1, and PD-L2 expression on CD14 + monocytes in active TB patients were elevated^27^. These data indicate the dynamics of PD-1 signaling during different phase of infection disease development.

Our data also indicated T-cell immune response was significantly inhibited in mice co-stimulated by MTB antigen and lung cancer antigen. Inactivated APCs, dysfunctional Tregs and memory T cells were involved in the reduced immune response in vivo. Thus we concluded blockade of PD-1/PD-L1 pathway may reverse the function loss in T-cell population in TB patients. A recent study showed immune checkpoint blockade directly inhibits the PD-1 pathway and results in a significant prevention of the brain from brain pathogens^28^. Although further pre-clinical studies are in need to validate this conclusion, our study provides proof of concept that he PD-1/PD-L1 pathway could be a host drug target for immunomodulatory treatments in the future.
